# SH003 represses tumor angiogenesis by blocking VEGF binding to VEGFR2

**DOI:** 10.18632/oncotarget.8808

**Published:** 2016-04-18

**Authors:** Hyeong Sim Choi, Min Kyoung Kim, Kangwook Lee, Kang Min Lee, Youn Kyung Choi, Yong Cheol Shin, Sung-Gook Cho, Seong-Gyu Ko

**Affiliations:** ^1^ Department of Science in Korean Medicine, Graduate School, Kyung Hee University, Seoul, Korea; ^2^ Jeju International Marine Science Center for Research and Education, Korea Institute of Ocean Science & Technology (KIOST), Jeju, Korea; ^3^ Department of Preventive Medicine, College of Korean Medicine, Kyung Hee University, Seoul, Korea; ^4^ Department of Biotechnology, Korea National University of Transportation, Jeungpyeong, Chungbuk, Korea

**Keywords:** SH003, tumor angiogenesis, VEGF, VEGFR2, TCM

## Abstract

Tumor angiogenesis is a key feature of cancer progression, because a tumor requires abundant oxygen and nutrition to grow. Here, we demonstrate that SH003, a mixed herbal extract containing *Astragalus membranaceus* (Am), *Angelica gigas* (Ag) and *Trichosanthes Kirilowii* Maximowicz (Tk), represses VEGF-induced tumor angiogenesis both *in vitro* and *in vivo*. SH003 inhibited VEGF-induced migration, invasion and tube formation in human umbilical vein endothelial cells (HUVEC) with no effect on the proliferation. SH003 reduced CD31-positive vessel numbers in tumor tissues and retarded tumor growth in our xenograft mouse tumor model, while SH003 did not affect pancreatic tumor cell viability. Consistently, SH003 inhibited VEGF-stimulated vascular permeability in ears and back skins. Moreover, SH003 inhibited VEGF-induced VEGFR2-dependent signaling by blocking VEGF binding to VEGFR2. Therefore, our data conclude that SH003 represses tumor angiogenesis by inhibiting VEGF-induced VEGFR2 activation, and suggest that SH003 may be useful for treating cancer.

## INTRODUCTION

Traditional Chinese herbal medicines have long been used to treat diseases including cancer, while effective chemical components have not been clearly elucidated [1–12]. SH003 is a mixed herbal extract containing *Astragalus membranaceus* (Am), *Angelica gigas* (Ag), and *Trichosanthes Kirilowii* Maximowicz (Tk), which is based on the principle of traditional Chinese medicine [12–16]. Each herbal component has been revealed to have anti-cancer effects, and our resent study has shown that a mixture of those components named SH003 was better than each component in anti-breast cancer effect [17–22].

Tumor angiogenesis is an essential process for cancer progression, as angiogenic vessels supply nutrients and oxygen to the tumor [23, 24]. Vascular endothelial cells (ECs) in the angiogenic process move toward the tumor and form new vessels [25, 26]. Vascular endothelial growth factor (VEGF) is mainly released from tumor cells and targets VEGF receptor 2 (VEGFR2), which is a crucial paracrine path for tumor angiogenesis [27]. VEGF-induced VEGFR2-mediated signaling highly expresses matrix metallopeptidase-9 (MMP-9), promoting a directional migration of ECs [28–30]. Therefore, targeting VEGF-induced VEGFR2-mediated signaling in ECs is important for cancer treatment [24, 31–33].

Our previous study found that SH003 reduced CD31-stained vessel numbers in tumor tissues [13], which raised a question of whether SH003 directly affects tumor angiogenesis. Ag inhibits VEGF-induced angiogenesis, while Am induces angiogenesis [18, 34–36]. In addition, an anti-angiogenic effect of Tk is not reported. Accordingly, we hypothesized that SH003 might have a therapeutic usage in inhibiting tumor angiogenesis.

Our *in vitro* and *in vivo* studies demonstrate that SH003 inhibits VEGF-induced tumor angiogenesis by blocking VEGFR2-mediated signaling, which suggest that metronomic SH003 treatment could be useful for treating cancer.

## RESULTS

### SH003 inhibits VEGF-stimulated human endothelial cell migration, invasion and tube formation

**Figure 1:**
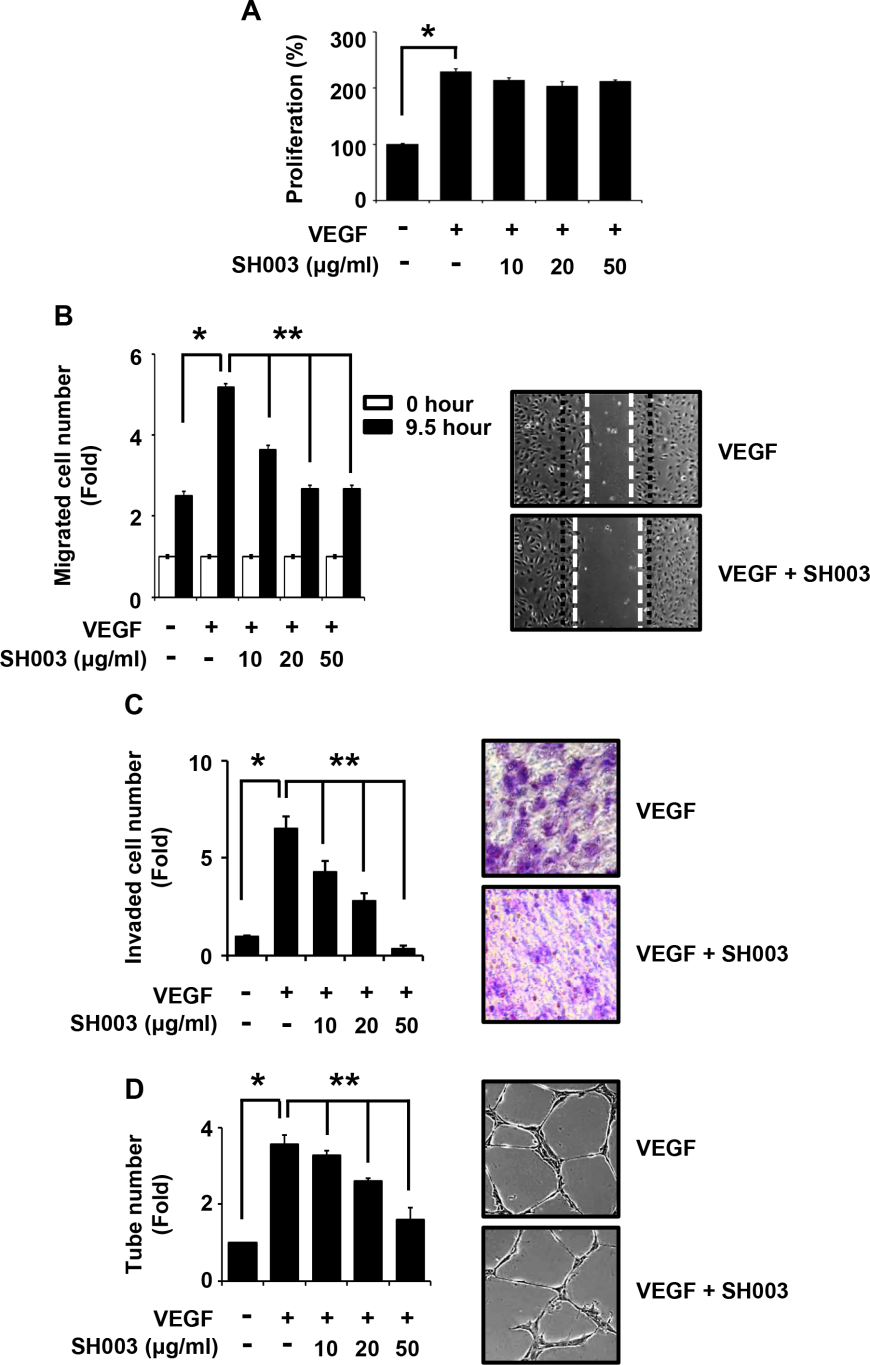
SH003 inhibits VEGF-induced angiogenic properties of HUVEC **A.** Cell viability in the presence of VEGF and different concentration of SH003 was measured by MTT assay (mean ± SD; *n* = 6). **B.** Cell migration. Left panel, SH003 inhibits VEGF-induced cell migration in wound healing assay. Right panel, representative images of left panel results (x40). **C.** Cell invasion. Left panel, SH003 inhibits VEGF-induced cell invasion in Boyden chamber assay. Right panel, representative images of left panel results (x40). **D.** Tube formation. Left panel, SH003 inhibits VEGF-induced tube formation in Matrigel. Right panel, representative images of left panel results (x40). * and **, *p* < 0.05.

We first investigated whether SH003 inhibits *in vitro* VEGF-dependent angiogenic processes in human endothelial cells such as proliferation, migration, invasion and tube formation, as those cellular aspects are basically investigated in the *in vitro* angiogenesis [33, 37–39]. To measure an effect of SH003 on the proliferation, human umbilical vascular endothelial cells (HUVECs) were treated with VEGF (50ng/ml) and different concentrations of SH003 (10, 20 or 50μg/ml) for 24 hours. While VEGF increased HUVEC proliferation rate as previously reported [38, 40], SH003 did not affect VEGF-induced HUVEC proliferation (Figure 1A). However, SH003 inhibited the number of VEGF-dependent migrated cells into the scratched region, when cells were treated with VEGF and SH003 for 9.5 hours (Figure 1B). Likewise, SH003 inhibited the HUVEC invasive ability induced by VEGF, as SH003 reduced invaded cell numbers in dose-dependent manner (Figure 1C). Moreover, SH003 inhibited VEGF-dependent tube formation on matrigels (Figure 1D). Therefore, our data suggest that SH003 effectively inhibits VEGF-dependent cell migration, invasion, and tube formation of the endothelial cells but not the proliferation.

### SH003 inhibits VEGF-induced tumor angiogenesis *in vivo*

**Figure 2:**
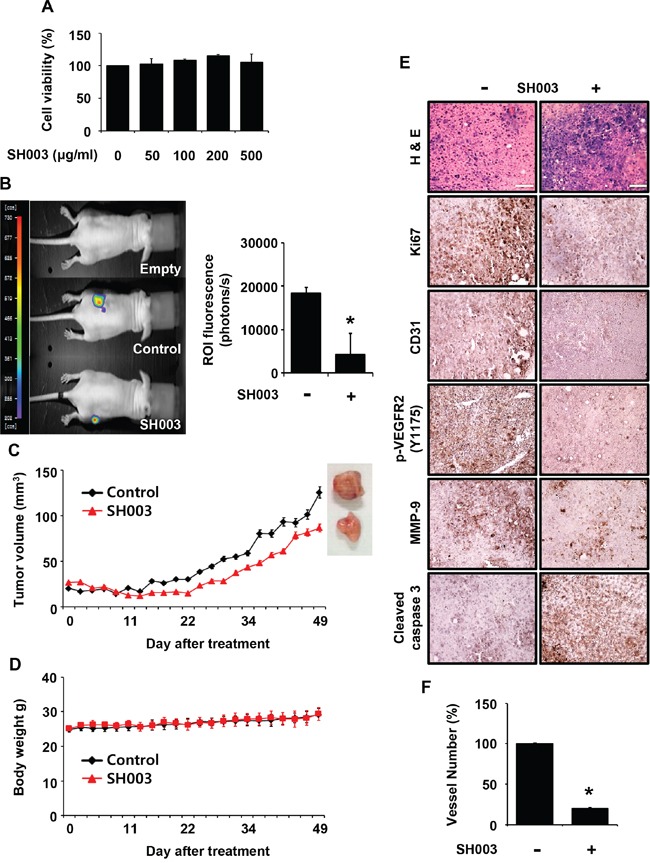
SH003 inhibits tumor growth *in vivo* **A.** The effect of SH003 on the viability of Panc-28-luc cells was determined by the MTT assay (mean ± SD; *n* = 6). **B.** Left panel, effects of SH003 on xenograft tumor growth were analyzed by bioluminescence imaging system. Right panel, bars represent quantitative data for left panel results. **C-D.** SH003 inhibits tumor growth without detectable toxicity. **E.** Immunohistochemistry of tumor sections. **F.** The number of CD31-positive vessels in tumor tissues were counted (*n;* control group = 7 or SH003 group = 4). *, *p* < 0.05.

Angiogenesis has a pivotal role in tumor growth and metastasis, and VEGF/VEGFR2-mediated signaling is crucial for tumor angiogenesis [28]. As our data showed that SH003 inhibited VEGF/VEGFR2-mediated angiogenesis *in vitro*, we further evaluated whether SH003 represses tumor growth by inhibiting tumor angiogenesis *in vivo*. SH003 did not inhibit the viability of Panc-28-luc pancreatic tumor cells *in vitro* (Figure 2A). However, SH003 retarded tumor growth *in vivo*, when Panc-28-luc cells were *s.c.* injected into the immunodeficient mice, and then added *p.o.* with SH003 (Figure 2B). Consistently, the average tumor volume was smaller in mice treated with SH003 than in the control (Figure 2C). In addition, SH003 did not affect body weights (Figure 2D), indicating that SH003 at the concentration we used might be safe. The immunohistochemistry confirmed that SH003 inhibited pancreatic tumor growth via tumor angiogenesis. SH003 reduced expression levels of Ki67, p-VEGFR2 and MMP-9, and increased expression level of cleaved caspase-3 in tumor tissues (Figure 2E). Moreover, when we stained tumor tissues with anti-CD31 antibody, and SH003 reduced the vessel numbers (Figure 2E and 2F). Therefore, our data indicate that SH003 suppresses pancreatic tumor growth by inhibiting tumor angiogenesis.

**Figure 3:**
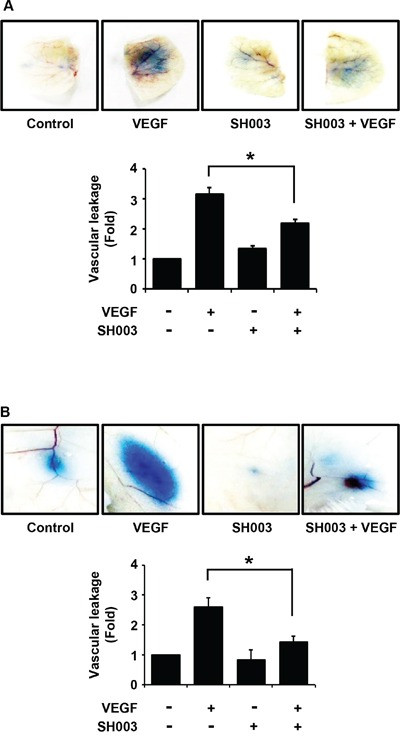
SH003 inhibits VEGF-induced vascular permeability *in vivo* **A.** Top panel, SH003 inhibits VEGF-induced leakage of Evans blue dye into ears of mouse (mean ± SD; *n* = 5). Bottom panel, data represent quantitative results for top panel results. **B.** Top panel, in back skins the effect of SH003 on the vascular permeability was determined by the leakage assay (mean ± SD; *n* = 5). Bottom panel, data represent quantitative results for top panel results. *, *p* < 0.05.

In addition, we investigated whether SH003 affects VEGF-induced vascular permeability *in vivo*. SH003 reduced VEGF-induced leakage of Evans blue dye in the ears or back skins of mice (Figure 3A and 3B), indicating that SH003 inhibits VEGF-induced vessel permeability *in vivo*.

### SH003 suppresses VEGF/VEGFR2-mediated angiogenic signaling by blocking VEGF binding to VEGFR2

**Figure 4:**
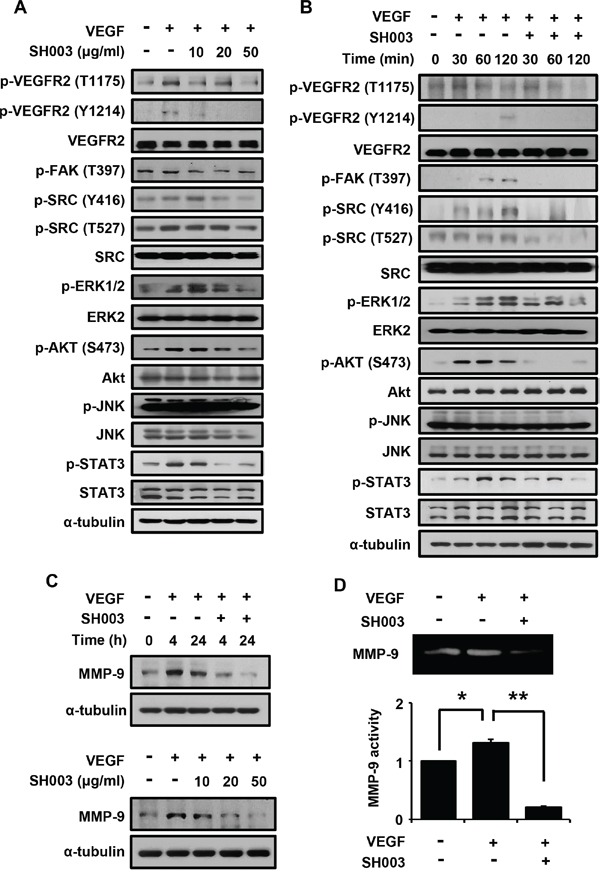
SH003 inhibits VEGF-induced signaling in the endothelial cells **A.** SH003 inhibition of VEGF-induced signaling was examined at different concentrations of SH003. **B.** SH003 inhibition of VEGF-induced signaling was examined for 120 minutes. **C.** SH003 inhibition of MMP-9 expression was examined at different time points or at concentrations of SH003. **D.** MMP-9 activity was measured by zymography assay. * and **, *p* < 0.05.

Next, we investigated SH003 effect on VEGF-dependent VEGFR2-mediated angiogenic signaling. SH003 inhibited VEGF-stimulated intracellular angiogenic signaling in dose-dependent manner, when HUVECs were pretreated with SH003 at different concentrations for 1 hour and then treated with VEGF for another 1 hour (Figure 4A). SH003 decreased VEGF-dependent VEGFR2 phosphorylation at T1175 and Y1214 residues, resulting in reduction of phosphorylation of FAK, SRC, ERK, AKT, and STAT3. Moreover, when we examined the time-course of SH003 inhibition of VEGF-induced VEGFR2-mediated signaling, SH003 strongly suppressed VEGF-dependent phosphorylation of VEGFR2, FAK, SRC, ERK, AKT, and STAT3 until 2 hours after VEGF stimulation (Figure 4B). Therefore, our data suggest that SH003 has anti-angiogenic effect via inhibiting VEGF-stimulated VEGFR2-mediated angiogenic signaling in the endothelial cells.

We next examined whether SH003 affects MMP-9 expression, as VEGF-induced tumor angiogenic vessel cells require MMP-9 to move toward a tumor mass [41, 42]. SH003 inhibited VEGF-induced MMP-9 protein expression in dose- and time-dependent manners (Figure 4C). Accordingly, SH003 inhibited VEGF-induced MMP-9 enzymatic activation (Figure 4D).

**Figure 5:**
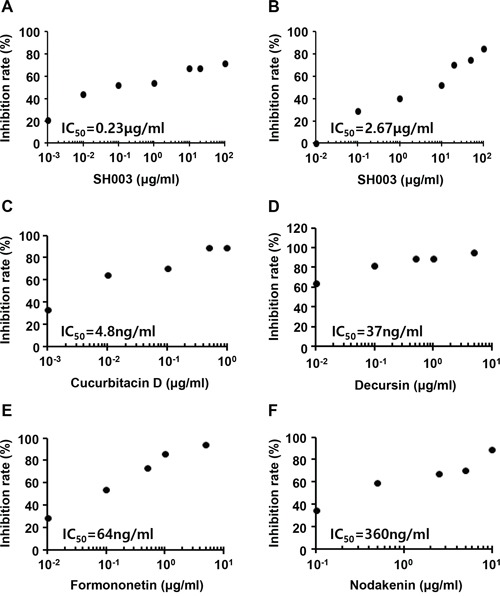
SH003 inhibits VEGF binding to VEGFR2 **A-B.** SH003 and btVEGF were treated on the plate where recombinant human VEGFR2 or VEGFR1 was coated. **C-F.** Cucurbitacin D, decursin, formononetin, or nodakenin together with btVEGF were treated on the plate where recombinant human VEGFR2 was coated.

As SH003 inhibited VEGF-dependent VEGFR2 phosphorylation and its downstream signaling, we further analyzed whether SH003 inhibits an interaction of VEGF with VEGFR2. In our *in vitro* VEGF-VEGFR2 interaction assays, SH003 directly inhibited the interaction between VEGF and VEGFR2 (Figure 5A). Moreover, SH003 inhibited the interaction between VEGF and VEGFR1, while the inhibitions showed different IC_50_ values (Figure 5B). We recently confirmed that SH003 and its herbal components contained cucurbitacin D, decursin, formononetin, and nodakenin [13]. Therefore, we further examined whether those chemical compounds inhibits VEGF binding to VEGFR2. While different IC_50_ values were determined, each chemical compound inhibited VEGF binding to VEGFR2 (Figure 5C–5F). Those data suggest that SH003 and its compounds directly inhibit VEGF/VEGFR2 interaction.

## DISCUSSION

VEGF activation of VEGFR2-mediated signaling is a key step for tumor angiogenesis [25, 27, 38, 43–45]. Therefore, the inhibition of VEGF/VEGFR2-mediated angiogenic signaling is one of excellent therapeutic ways to treat cancer growth and/or metastasis [45–47]. In this study, we found that SH003 inhibited tumor angiogenesis by blocking VEGF binding to VEGFR2.

Our previous study showed that SH003 at higher concentrations caused breast cancer cell death by inhibiting STAT3-mediated signaling pathway [13]. SH003 concentrations tested in this study was lowered than those in the previous study [13], as we considered the metronomic approach to target tumor angiogenesis [48–50]. SH003 significantly inhibited VEGF-induced angiogenic processes *in vitro* including endothelial cell migration, invasion and capillary-like structure formation with no effect on the proliferation. In addition, SH003 at the concentration in this study did not affect pancreatic cancer cell proliferation. Thus, it guarantees that SH003 is not toxic but effective in the treatment of tumor angiogenesis. Moreover, our *in vivo* data showed that SH003 inhibited xenograft tumor growth as well as VEGF-stimulated vascular permeability. Therefore, our *in vitro* and *in vivo* data strongly suggest that SH003 has anti-angiogenic effect.

SH003 blocked VEGF-induced phosphorylation of VEGFR2 by directly interrupting VEGF binding to VEGFR2, resulting in the inhibition of downstream signaling. Moreover, we revealed that components in SH003 inhibited VEGF/VEGFR2 interaction. While decursin has been revealed to inhibit VEGFR2 phosphorylation [33], this study first shows that chemical components including decursin have abilities to block VEGF binding to VEGFR2. However, it still remains to be elucidated how many chemical components in SH003 interfere with the interaction between VEGF and VEGFR2, and how those components work together as a whole. Meanwhile, we found that SH003 inhibited VEGF binding to VEGFR1, although SH003 concentrations for the inhibition of VEGF/VEGF1 binding were higher than those for the inhibition of VEGF/VEGFR2 binding. Thus, SH003 effect on VEGF binding to either VEGFR2 or VEGFR1 suggests that SH003 could be applied to other vascular diseases beyond tumor angiogenesis.

This study first revealed that SH003 as the modified version of TCM inhibits tumor angiogenesis by directly inhibiting VEGF/VEGFR2 interaction. As the concentration of SH003 in anti-angiogenesis role was lower than that in anti-breast cancer function, we suggest that metronomic treatment of SH003 with particular anti-cancer agents could be effective to treat cancer development and metastasis, especially pancreatic cancer. Our ongoing studies include what effective molecules directly inhibits VEGFs/VEGFRs interactions, which will serve better knowledge for TCMs and help understanding vascular diseases.

## MATERIALS AND METHODS

### Preparation of SH003 extacts

SH003 was prepared as described previously [13]. In brief, each component was provided from Hanpoong Pharm and Foods Company (Jeonju, Korea) manufactured by the Good Manufacturing Product (GMP) and mixed as follows: 333g of *Astragalus membranaceus*, 333g of *Angelica gigas*, and 333g of *Trichosanthes Kirilowii* Maximowicz. The mixture was extracted by 30% ethanol and then stored at −80°C until use. Table 1 shows information on SH003 ingredients.

**Table 1:** Crude components and amounts of SH003

Scientific name	Latin name	Chinese name	Amount (g)
*Angelica membranaaceus*	Astragali Radix	黃芪	333.0
*Angelica gigas*	Angelicae Gigantis Radix	當歸	333.0
*Trichosanthes Kirilowii* Maximowicz	Trichosanthis Fructus	天花粉	333.0

### Cell cultures

Human umbilical vein endothelial cells (HUVECs) were kindly provided by Dr. Kwang Seok Kim (Korea Institute of Radiological and Medical Sciences, Seoul, Korea) and cultured in endothelial cell medium (ECM) with 5% fetal bovine serum, 1% endothelial cell growth supplement, and 1% penicillin/streptomycin solution. Panc-28-luc-luc cells were kindly provided by Dr. Bharat B. Aggarwal (UT-MDA, Houston, USA) and cultured in DMEM with 10% fetal bovine serum and 1% penicillin/streptomycin.

### Proliferation, scratching, tube formation, and invasion assay

For proliferation assays, HUVECs were seeded at 5×10^3^ cells in 96-well plates and then exposed to various concentrations of SH003 in the presence or absence of VEGF (50ng/ml). 5×10^3^ Panc-28-luc cells were plated onto 96-well plates and treated with different concentrations of SH003. Cells were incubated for 24 or 72 hours and the cell viability was measured by MTT assay. For scratching assay, HUVECs were plated onto 12-well plates, scratched, and then washed with phosphate-buffered saline (PBS). After treatment with SH003 and VEGF (50ng/ml) for 9.5 hours, cells migrated toward the wound region were counted. For *in vitro* tube formation assay, 8×10^4^ HUVECs were plated onto matrigel-coated 12-well plates, and treated with VEGF (50ng/ml) and various concentrations of SH003. After incubation for 9 hours, cells were fixed with 4% paraformaldehyde and capillary-like structures were measured. For invasion assay, 6×10^4^ HUVECs were plated onto matrigel-coated 8μm pore size chambers. The upper chambers were filled with different concentrations of SH003 and the bottom well was added VEGF (50ng/ml) as the chemoattractant. After incubation for 5~7 days, the invaded cells were fixed with 4% paraformaldehyde and stained with 0.05% crystal violet. Crystal violet-positive invaded cells were counted. Each experiment was performed in triplicate and repeated three times.

### *In vivo* studies

All animal experiments were approved by Kyung Hee University Institutional Animal Care and Use Committee (KHU-IACUC). Five-week-old male Balb/c nude mice were purchased from Jungang Lab Animal Inc. (Seoul, Korea). For vascular leakage assays, SH003 (20μg) was injected into the ears or back skins in the presence or absence of VEGF (100ng) for 30 min. 150μl of 1% Evans blue dye was injected via tail vein to detect VEGF-induced vascular leakage. The stained tissues were removed and weighed. Evans blue was extracted from the tissues with 100μl of formamide solution for 24 hours at 55°C and measured spectrophotometrically at 610 nm. For xenograft tumor growth assay, Panc-28-luc cells (1×10^6^) mixed with matrigels were subcutaneously implanted. For the *in vivo* bioluminescence imaging analyses, mice were randomly separated into three groups (background, control, and SH003). SH003 (2mg/kg) was orally administrated to SH003 group, while saline to control group. Mice were injected with 200μl D-luciferin using 25G syringes and incubated for 60 minutes. The image was captured in NightOWL LB 983 and analyzed using Indigo program (Berthold Technologies, Bad Wildbad, Germany). Body weights and tumor volumes were measured three times a week. Tumor volumes were determined by a formula: volume = length × width^2^ × 0.5. At the end of experiments, mice were euthanized and then tumors were isolated. For histological analyses, tumors were fixed with 4% paraformaldehyde and embedded in paraffin. Immunohistochemical staining for Ki-67, CD31, p-VEGFR2 (Y1175), and cleaved caspase-3 were carried out.

### Western immunoblotting

Total 20μg of protein was loaded on 10~15% SDS-PAGE, transferred to nitrocellulose membrane and blotted by appropriate antibodies. Anti-p-JNK, -ERK2, and -p-AKT (S473) antibodies were purchased from Santa Cruz Biotechnology (Santa Cruz, CA, USA). Anti-AKT, -p-ERK1/2, -JNK, -p-VEGFR2 (Y1175), -MMP-9, -VEGFR2, -p-FAK (T397), -p-SRC (Y416), -p-SRC (Y527), -SRC, -p-STAT3, and -STAT3 antibodies were obtained from Cell Signaling Technology (Danvers, MA, USA). Anti-p-VEGFR2 (Y1214) antibody was purchased from R&D systems (Minneapolis, MN, USA). Anti-α-tubulin antibody was purchased from Sigma (St. Louis, MO, USA).

### Zymography assay

For gelatinolytic activities of samples, cells were treated with VEGF and SH003 for 24 hours and then subjected to zymography. After incubation, medium was collected and concentrated using Amicon Ultra-4 centrifugal filters (Millipore, New York, USA). The concentrated was mixed with non-reducing 5X sample buffer and electrophoresed in 8% SDS-PAGE gels containing 0.2% gelatin. Gels were run at 90V for 3 hours at 4°C and washed for 40 minutes in 2.5% Triton X-100 solution at room temperature. The gels were incubated for 20 hours at 37°C in 50mM Tris-HCl containing 0.15 M NaCl and 10 mM CaCl_2_ (pH 7.8). Next, the gels were stained for 1 hour in 0.05% Coomassie Brilliant Blue solution and de-stained until clear bands were visible.

### *In vitro* solid-phase binding assay of biotinylated VEGF to recombinant human VEGFR1-2

The method was performed as described previously [51]. 96-well microplate (Thermo Fisher Scientific, Waltham, USA) was coated with 100μl of phosphate buffer saline (PBS) containing 500ng/ml of either VEGFR-1 or -2 ECD/Fc chimera (R&D Systems, Minneapolis, USA). The plate was sealed and incubated overnight at 4°C. After 3 times washes with 200μl of PBS containing 0.05% (v/v) Tween 20, the plate was blocked by adding 100μl of PBS with 1% (w/v) bovine serum albumin (BSA), and incubated for 2-3 hours at room temperature. The plate was washed 3 times and added with 100μl of diluted standards (biotinylated VEGF (btVEGF), R&D systems, Minneapolis, USA) or compounds (with 50ng/ml btVEGF) in PBS. After 2.5-3 hours incubation at room temperature, the plate was washed 3 times, and 100μl of streptavidin-HRP (R&D Systems, Minneapolis, USA) was diluted at 1:250 in blocking buffer. The plate was incubated for another 1 hour at room temperature, and then washed five times with 200μl of wash buffer with 100μl of substrate solution (BD Biosciences, Sandiego, USA). After 1-3 hours incubation at room temperature, the plate was then added with 50μl of stop solution (1M H_3_PO_4_) to each well. The signal measured at 450 nm using ELISA plate reader.

### Statistical analysis

All experimental data were presented as a mean ± SD and analyzed by Student *t*-test or one-way ANOVA using SPSS software. *P*-value < 0.05 means statistically significant.
